# Evaluating machine learning techniques for archaeological lithic sourcing: a case study of flint in Britain

**DOI:** 10.1038/s41598-021-87834-3

**Published:** 2021-05-13

**Authors:** Tom Elliot, Robert Morse, Duane Smythe, Ashley Norris

**Affiliations:** 1 Department of Archaeology, Classics and Egyptology, University of Liverpool, 12-14 Abercromby Square, Liverpool, L69 7WZ UK; 2 Intelligent Ultrasound, Floor 6A, Hodge House, 114-116 St Mary Street, Cardiff, CF10 1DY UK; 3 Department of Earth Sciences, South Parks Road, Oxford, OX1 3AN UK; 4 Norris Scientific, PO Box 812, Kingston, TAS 7050 Australia

**Keywords:** Characterization and analytical techniques, Geochemistry, Statistics

## Abstract

It is 50 years since Sieveking et al. published their pioneering research in *Nature* on the geochemical analysis of artefacts from Neolithic flint mines in southern Britain. In the decades since, geochemical techniques to source stone artefacts have flourished globally, with a renaissance in recent years from new instrumentation, data analysis, and machine learning techniques. Despite the interest over these latter approaches, there has been variation in the quality with which these methods have been applied. Using the case study of flint artefacts and geological samples from England, we present a robust and objective evaluation of three popular techniques, Random Forest, K-Nearest-Neighbour, and Support Vector Machines, and present a pipeline for their appropriate use. When evaluated correctly, the results establish high model classification performance, with Random Forest leading with an average accuracy of 85% (measured through F1 Scores), and with Support Vector Machines following closely. The methodology developed in this paper demonstrates the potential to significantly improve on previous approaches, particularly in removing bias, and providing greater means of evaluation than previously utilised.

## Introduction

Identifying the geological source of lithic materials is a central aim of research into early prehistoric societies^1,2^. In addition to providing a simple link between the location of discovery and the geological origin of stone materials or artefacts, sourcing studies have an important role in assisting the development of archaeological theory and interpretation. This includes identifying and evidencing connections between disparate archaeological sites based on similar materials^3–5^, improving understanding of the technological processes involved in tool manufacture^6,7^, establishing potential social and trade networks, and offering insight into perceptions of the physical environment at specific places in prehistoric landscapes^8,9^.

Traditionally, the underlying premise of sourcing studies has been the provenance postulate^10^. This states that for lithic sourcing to be successful, the variation between sources geochemically must be larger than that within them. Whilst this has been a reasonable *a-priori* proposition in previous years, arguably this has overemphasised the importance of variation between sample data, at the expense of looking for appropriate techniques to investigate the structure inherent within the data itself.

With the advent of Machine Learning techniques, such separation is routinely possible, using iterative methodologies that improve on their results through validation of reliable training data. The utility of such approaches has been seen more widely in Archaeology, including towards remote sensing and prediction or classification of archaeological sites^11–13^, the recording and creation of artefact typologies^14–19^, and more recently for lithic sourcing ^20–24^. For this latter topic, these techniques promise more powerful approaches to the separation of geological samples and increased accuracy over classical statistical techniques. However, without appropriate sampling, pipeline development, and evaluation, these methods are likely to propagate errors rather than reduce them, misleading researchers as to the validity of their results. There is therefore increasing need for effective and appropriate ways to use these techniques and to evaluate their use in lithic sourcing.

Despite widespread documentation on the correct usage of these techniques, several problems can be identified in the recent literature on lithic sourcing. These include basic prerequisites such as inadequate sampling from individual geological sources for machine learning techniques to effectively learn from^23^, to perhaps more importantly, a large number which use classification techniques with no ‘none of the above’ or ‘other’ class or method to discriminate from the geological sample sites used to compare artefacts with^20–22,24,25^. The failure to create such a class or method for the model to use can lead to false positive results (type I errors), allowing no option to rule out the geological sample sites used. Given these issues, it is important to stop and reflect on the way these models are generated, before wider interpretation of the results are used to make significant archaeological claims, as increasingly faced with other approaches in archaeological science^26^.

**Figure 1:**
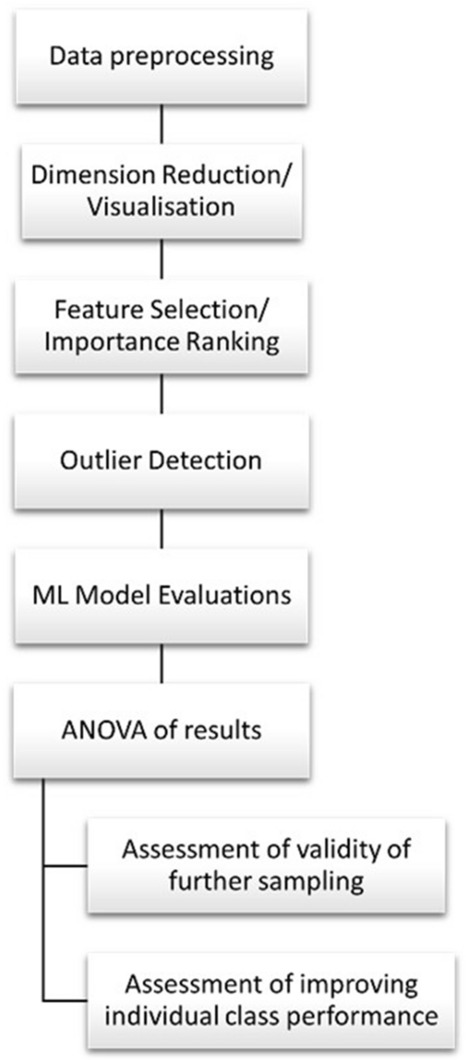
Flowchart of pipeline.

The aim of this research was to robustly evaluate the accuracy of three popular Machine Learning techniques towards their classification of geological samples of flint from England and Wales, as well as demonstrate the correct use of these approaches. We present a robust pipeline of; data pre-processing, dimension reduction and visualisation, feature selection and importance ranking, outlier detection, model evaluation, and analysis of the final results. Finally, efforts to improve these results and those of the individual classes (the geological sample sites) are evaluated to identify strategies for future research. The results of this paper will then be used to provide sourcing determinations for analysed artefacts, to be published more fully in future. A flow chart of this pipeline is shown in Fig. 1.

The techniques investigated were Random Forest, Support Vector Machine, and K-Nearest Neighbour^27^. These are supervised classification algorithms which use different methods to map unknown data to pre-established classes of known data. Random Forest uses large numbers of randomised decision trees to differentiate data based on their values, Support Vector Machines look to optimise the margin between groups of data before classification, and K-Nearest Neighbour assigns unknown data based on the frequency of the classes of surrounding data. All models generated were trained on the optimum features as determined by feature selection and feature importance processes prior to evaluation.

## Methods

The pipeline to evaluate the machine learning models used in this paper was constructed in Jupyter-lab (https://jupyterlab.readthedocs.io/en/stable/), using Python v3.7.1 (https://www.python.org). The computationally expensive hyperparameter optimisation and model validations were carried out using a Linux virtual machine hosted on the cloud computing platform Microsoft Azure, utilising 8 GB of RAM and 4 CPUs. The following Python libraries were used: Numpy v1.15.3^28^, Scikit-learn v0.20.1^29^, Pandas v0.23.4^30^, Matplotlib v3.0.2^31^, Seaborn v0.9.0^32^, Swifter v0.287^33^ and Imbalanced-learn^34^.

### General modelling details

All model performances were evaluated using the unweighted macro F1 score^35,36^. The F1 score is the harmonic mean of the precision and recall. The models were evaluated by taking the unweighted average of all class specific F1 scores. This was chosen to avoid making the overall F1 score bias to the more numerous classes, as this would give a false confidence in the model performance.

$$precision= \frac{tp}{tp+fp}$$

$$recall= \frac{tp}{tp+fn}$$

where

$$tp=true \,postives$$

$$fp=false \,positives$$

$$fn=false \,negatives$$

F1 therefore equals:

$$F1=2*\frac{precision*recall}{precision+recall}$$

### Geological sample collection

The data for this research forms part of the dataset produced by the primary author^21^ as part of doctoral research into the source of 118 Mesolithic artefacts from 12 archaeological sites in the Lower Wye Valley region, on the Anglo-Welsh border. The geological samples from this dataset totalled 532 samples from 414 nodule fragments, collected from 21 bedrock and 14 superficial deposit geological samples sites from England and Wales. Tables 1 and 2 summarise the bedrock and superficial geological sample sites used in this research. Several sample sites were not included for this research either due to inadequate numbers of samples or problematic provenance (such as the superficial deposit material from Brandon Country Park, used to represent bedrock geology in the thesis).

**Table 1:** Table listing bedrock geological sample sites used in this research (based on Elliot 2019).

Bedrock sample site name	Site code
Flamborough Head	FH
Enthorpe Railway	ER
Welton Wold Quarry	WW
Trimmingham Cliffs	TC
Caistor St Edmund Quarry	CS
Kensworth Quarry	KQ
Aston Rowant Nature Reserve	AR
South Lodge Chalk Pit	SL
Fognam Quarry	FG
Winterbourne Chalk Pit	WB
Boxford Chalk Pit	BX
Pewsey Farm	PF
West Harnham	WH
Shillingstone Quarry	SQ
White Nothe	WN
Beer Head	BH
Peacehaven Steps	PH
Langdon Bay	LB

**Table 2:** Table listing superficial deposit geological sample sites used in this research (based on Elliot 2019).

Superficial deposit sample site	Site code	‘Region’	‘Region’ code
Aber Mawr	AB	NA	NA
Llanvihangel Crucorney	LV	NA	NA
Blackstone Rocks	BR	Severn Valley/Severn Estuary	SV_SE
Sandhurst Hill	SH		
Cumberland Farm	CF		
Bushley Green	BG		
Keynsham	KY	Bristol Avon	BA
Boundary Farm	BF		
Cropthorne	CR	Warwickshire Avon	WA
Green Hill	GH		
Aston-on-Carrant	AC		
Paxford	PX	Moreton-in-Marsh	MM
Woodhills Farm	WF		

### LA-ICP-MS methodology and data preparation

All samples were analysed in triplicate by LA-ICP-MS. This resulted in 1597 measurements, with 53 elements recorded for each. The instrumentation was a NewWave NWR213 Laser Ablation instrument (213 nm) and Perkin Elmer NexION 300Q quadrupole mass spectrometer. The carrier gas used was Helium. Dwell time for the geological samples was 40-60 s, and washout time 60 s. ^28^Si was used as the internal standard. NIST SRM610 was used as the external material standard and NIST SRM612 was analysed as an additional check, but not used for calibration. Data reduction was carried out in GEMOC/CSIRO GLITTER and Norsci LADR v0.6, with results normalised to 100% ^28^Si. Outliers exceeding two times the standard deviation from the mean average for each feature (element) and missing values were imputed with the mean average for that feature. No transformation of the data was conducted after this, other than scaling for use with SVM and KNN models.

### Pre-processing

#### Dimension reduction and visualisation using t-SNE

Prior to evaluating the machine learning techniques, it was first necessary to visualise the geochemical structure of the data between the geological samples. To do this, dimensionality reduction was first used. Dimensionality reduction techniques, such as Principal Component Analysis (PCA)^37^ and Linear Discriminant Analysis (LDA)^38^, allow the visualisation of the underlying structure of multivariate data by converting high dimensional data into two or three dimensions that can be viewed graphically. Such techniques aim to preserve as much information as possible from the original data within the resulting lower dimensional space. For this research, t-distributed Stochastic Neighbour Embedding (t-SNE)^39^ was chosen due to its greater capability over classical statistical techniques such as PCA. t-SNE projections give information on the similarity between groups of data points based on the structure of their mapping. Data points within clusters are more similar when compared to data points between clusters. This can be used to identify groups of geochemically similar samples. By colour-coding the data, the distinctness between them can be visually inspected and any outliers identified.

Both geological datasets were analysed to gain insight into the structure of the data. The bedrock data was grouped, then colour coded by sample site, while the superficial deposit sample sites were first grouped into geologically related ‘regions’ due to limited samples from some of these sites, then colour coded. The t-SNE utilised 10,000 iterations. All other parameters for the t-SNE were the default settings in the Python libraries used (see "Machine learning model evaluation" section below). The resulting t-SNE plots are shown in Fig. 1.

#### Outlier detection

A limitation in using classification techniques for lithic sourcing is the artificial creation of classes by which to group the geochemical data. This may be problematic for geologies such as flint, where the underlying structure of the data may be gradual in how it differs across long distances, due to the nature of its formation in large ocean environments. While the creation of these classes is necessary for classification techniques to be used, these must be based on the spatial and stratigraphic properties of the samples collected, such as the grouping of multiple flint nodules in a band, or multiple bands into a single sample site and so on. The key issues are the adequacy of sampling quantity, and the point at which these groups should be further separated into different classes based on inspection of the data.

Related to this, and as discussed above, if no means of detecting outliers from these groups are created alongside these artificial classes, the potential arises for artefacts under analysis to be incorrectly classified to these groups, committing a type I error in the generation of a false positive result. To avoid this error, the Local Outlier Factor model^40^ was used. The Local Outlier Factor model (LOF) can classify observations as outliers given examples of inliers and outliers. To do this the LOF model was fitted to the geological sample data, then used to predict the artefact samples as either inliers or outliers. Outliers were classified into the site ‘other’, while inliers were carried forward to be classified by the final model. As the purpose of the paper is to evaluate the machine learning techniques, the results of the artefact determinations and their archaeological significance will be published in future.

#### Feature selection and importance

To optimise the models, and to identify and evaluate the predictive power (importance) of the features analysed within the bedrock dataset, Recursive Feature Elimination with Cross-Validation (RFECV) was used^41^. RFECV iteratively builds models from the data one at a time. After each model is generated, the feature with the lowest predictive power is removed. Each model is then evaluated to identify the set of features that give rise to the best model. This minimises both physical and computational effort for future research by identifying which features decrease model performance through the addition of noise and allows for the most parsimonious model to be produced.

In this research, Random Forest models were used to identify these features^42^. The metrics for evaluating the features to be removed was Feature Importance. Each model was evaluated by threefold stratified cross-validation and using the F1 score. The mean averages for each stage of RFECV were visualised against all the feature selections. The feature combination with the highest F1 score was then chosen for all subsequent models.

### Machine learning model evaluation

**Figure 2:**
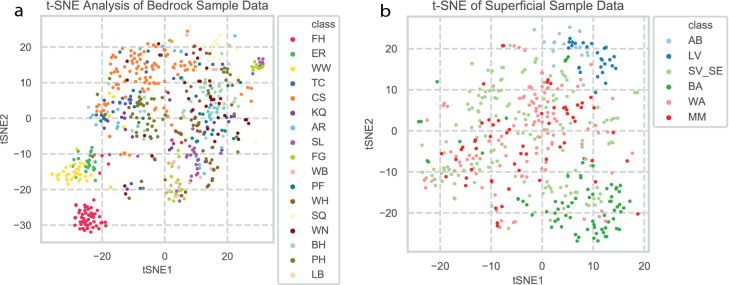
t-SNE plots of geochemical data. (**a**) Plot shows t-SNE coordinates for bedrock source coloured by location. (**b**) Plot shows t-SNE coordinates for superficial source coloured by location.

Once the data had been pre-processed to select the most useful features and to remove outlier data, the different models were trained and evaluated using 100-fold cross-validation to compare performances. The bedrock geological dataset was split randomly into 80% training and 20% testing data 100 times. This randomised process was stratified so that the proportions of samples within classes in the training data were representative of the entire dataset. Hyperparameter optimisation was done on the training data by 5-fold stratified cross-validation^43^. The original 80% training data fold was then used as input into a model, which was configured with the optimum hyperparameters using random grid search (see Supplementary Table 1). The models were then evaluated by F1 score by comparison of the predictions against the testing data class labels. The model performances were also compared by the visualisation of the 100 weighted-F1 scores in boxplots, shown in Fig. 2.

## Results

### Dimension reduction and visualisation results

As discussed above, the first step to evaluate the machine learning techniques was first to interrogate the structure of the geochemical data using t-SNE. The bedrock samples (Fig. 2a) showed greater clustering, consistent with the structured nature of the bedrock locations sampled. As might be expected, given the bias in bedrock sampling towards southern England, sites in northern England appear more distinct (see Flamborough Head (FH), Welton Wold (WW)). In contrast, the superficial deposit samples showed more limited structure (Fig. 2b). This is primarily due to the nature of their sampling, as no separation based on properties such as colour or inclusions was conducted. Further grouping based on these properties would likely have helped the models to differentiate between the materials from the different bedrock sources represented in these deposits. Grouping of multiple sample sites together from different deposits additionally removed any ability to isolate these materials, reducing accuracy. Future attempts to differentiate these materials and increased sampling would likely assist with this. Based on these visualisations and their interpretation, only the bedrock samples were used to evaluate the machine learning techniques.

### Outlier detection results

**Figure 3:**
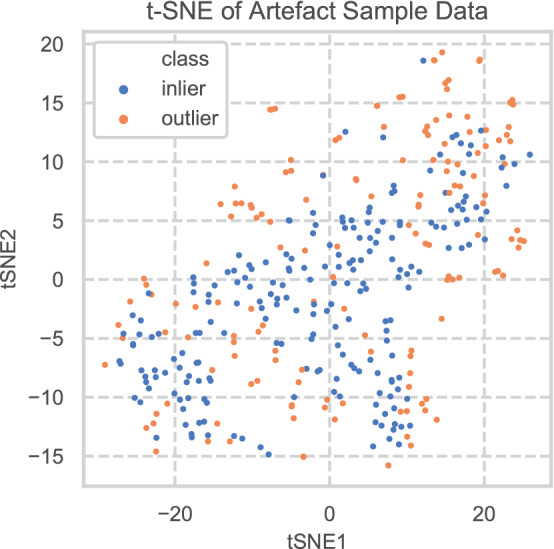
t-SNE plot showing identity of outliers identified by local outlier factor model.

The t-SNE mapping of the artefact data and their outlier status is shown in Fig. 3. The plot shows a substantial number of artefact analyses can be identified as outliers from the geological data the LOF model was fitted on, suggesting a range of sources for these materials. Further details of these results will be published in future.

### Feature selection and importance results

**Figure 4:**
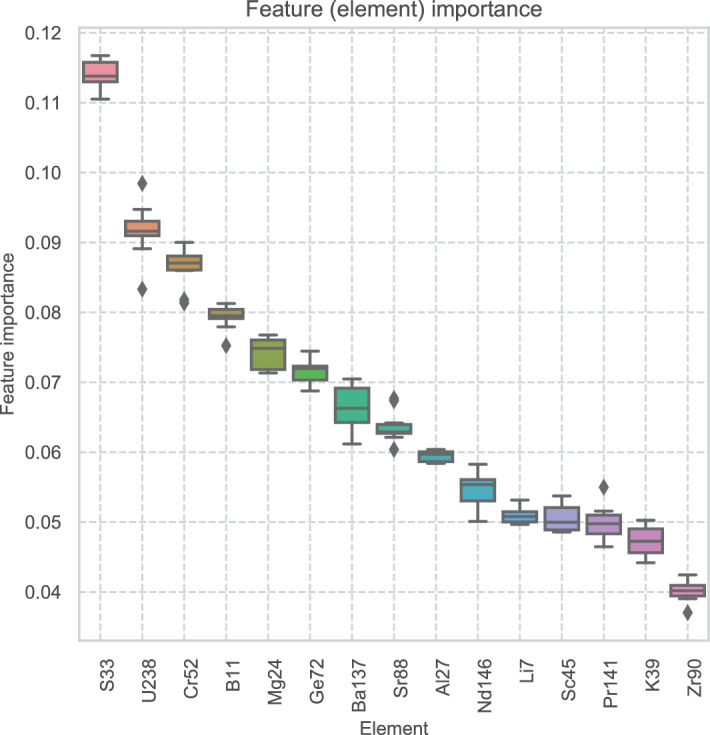
Feature importance results from RFECV.

**Figure 5:**
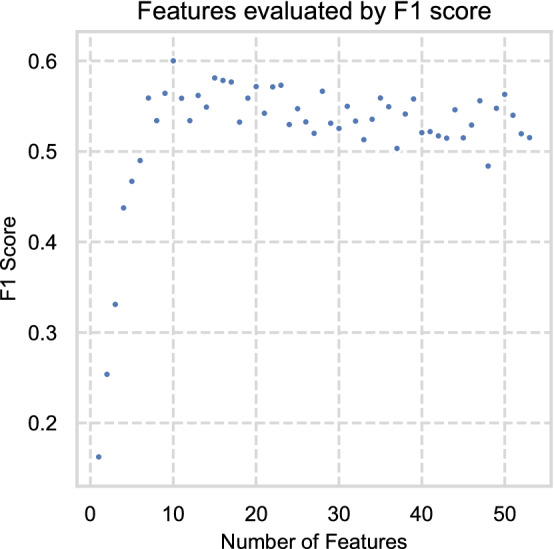
Scatter plot showing F1 scores for models built on features selected by recursive feature elimination.

The results of RFECV are shown in Fig. 4 and Fig 5 and Table 3. The model with the highest F1 score was built with 15 features (elements). In order of importance, these were ^7^Li, ^146^Nd, ^137^Ba, ^88^Sr, ^72^Ge, ^55^Mn, ^52^Cr, ^51^V, ^90^Zr, ^238^U, ^24^Mg, ^27^Al, ^39^K, ^11^B, and ^33^S. Additional features other than these decreased model performance by introducing noise. These features were then used for the model evaluations below. These results compare favourably with existing research^20,44–49^, with some differences likely due to analytical methods and sample locations used.

**Table 3:** Table of the top 20 features ranked.

Features	RFECV ranking
Zr90, Ba137, Sr88, Ge72, Cr52, S33, U238, Al27, B11, Mg24	1
Nd146	2
Sc45	3
K39	4
Pr141	5
Li7	6
V51	7
P31	8
Mn55	9
Cd111	10
La139	11

The results show much greater similarity to those of Brandl^20^ in particular, likely due to their more careful sampling, and the similarity between instrumentation and date of research. In particular, Brandl et al. found Strontium (Sr), Aluminium (Al), Magnesium (Mg), Manganese (Mn), Germanium (Ge), Rubidium (Rb) to be the most useful. Further information of exploratory data analysis of the dataset used here will be published in future in more detail.

### Model evaluation results

**Figure 6:**
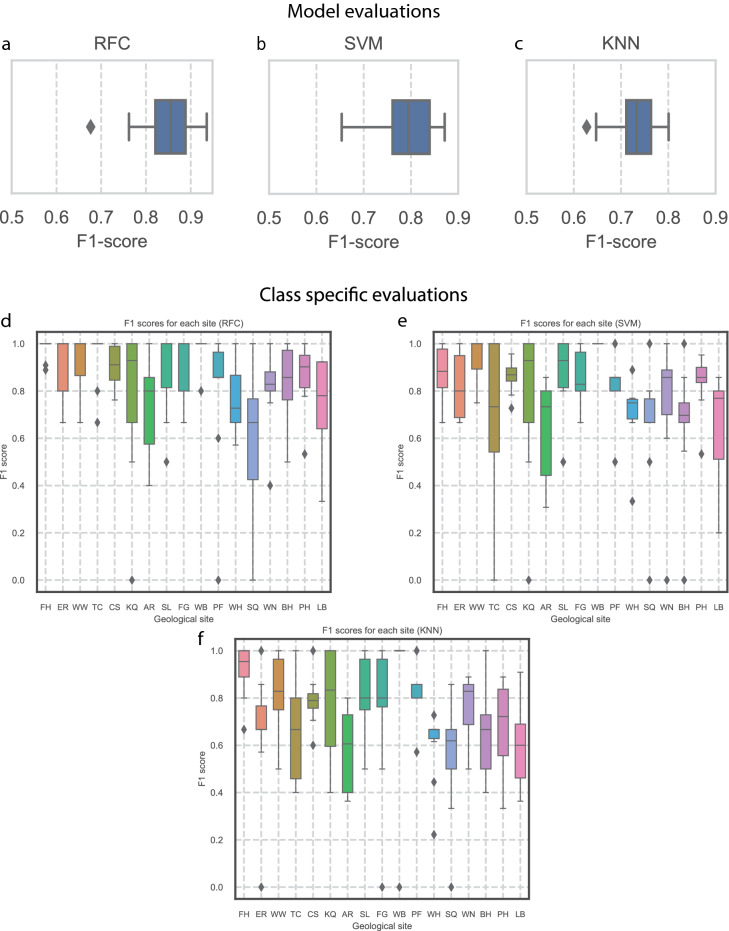
Evaluation of machine learning classifiers. (**a**–**c**) Distribution of F1 scores from 100-fold cross-validation for Random Forest, Support Vector Machine and K nearest neighbours, respectively. (**d**–**f**) Distribution of F1-scores for each source for Random Forest, Support Vector Machine and K nearest neighbours, respectively.

As seen in Fig. 6a–c, the Random Forest classifier outperformed both the Support Vector Machine and K-Nearest Neighbour models, with an overall or average F1-score of 0.85 (85%), compared with 0.79 (79%) for Support Vector Machines, and 0.73 (73%) for K-Nearest Neighbour respectively.

### Class specific evaluations

The results of the class specific F1-scores (as seen in Fig. 6d–f, and Table 4) likely demonstrate that some flint geologies are more distinct than others, that further separation of the different bands of flint or stratigraphic units is needed, or that greater sampling is needed at certain sites for individual performances to increase (discussed below). It is likely a combination of all three that would be needed to further refine the models. This can be more clearly understood by looking at the worst performing site, Shillingstone Quarry (SQ) where the poor performance is likely due to the limited number of samples from the site (n = 7 nodules from two stratigraphic locales), as well as understanding of the material properties of the flint, which included poorly-formed semi-tabular to lenticular nodules from weakly consolidated deposits^50^. This can be compared to better performing sites, such as Flamborough Head (FH) and Winterbourne (WB), both of which feature well-formed nodules from more consolidated Chalk and more samples. As most of the sites investigated represent a range of stratigraphically separate deposits grouped together to form a single class, it is likely that further separation alongside increased sampling would assist in improving class-specific scores.

**Table 4:** Class specific median F1 scores from the random forest classifier.

Site code/class	Median F1 score
FH	1
ER	1
WW	1
TC	1
CS	0.910973085
KQ	0.928571429
AR	0.8
SL	1
FG	1
WB	1
PF	0.857142857
WH	0.727272727
SQ	0.666666667
WN	0.828571429
BH	0.857142857
PH	0.902255639
LB	0.78030303

### ANOVA analysis of F1 scores

The last step was to assess the statistical difference in results between the models. This was conducted through one-way analysis of variance (ANOVA)^51^, using python modules Statsmodels^52^ v0.12.2, and Scipy^53^ v1.6.1. This was done by taking the median F1 scores of the 10-fold cross validation models for every geological site and comparing these between each ML method (n = 48). This revealed a significant difference between the model results (p < 0.002, alpha = 0.05). These results are presented in Table 5.

**Table 5:** Results of ANOVA comparison between ML method results.

	Sum of squares	df	Mean square	F	p-value
ML_method	0.163310	2.0	0.081655	6.815376	0.002481

**Figure 7:**
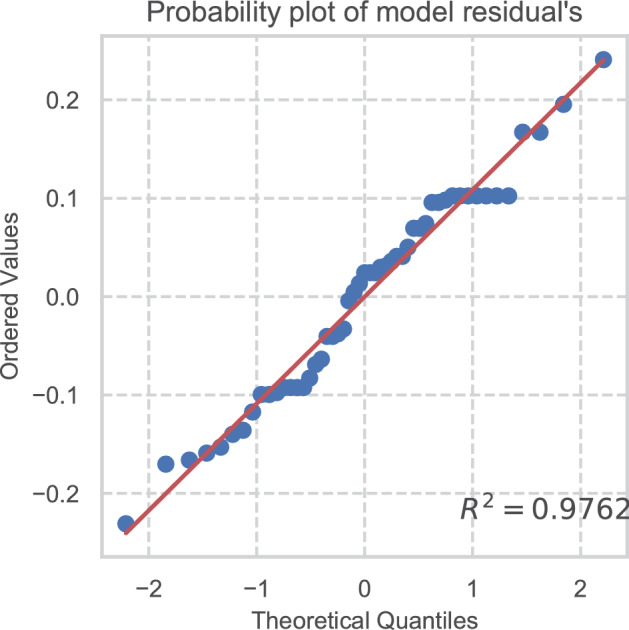
Probability plot of ordered values versus theoretical quantiles.

To confirm the test’s assumptions, a Shapiro–Wilk’s test^54^ was conducted, demonstrating normality of the median scores of the models (W = 0.97, p 0.29), and was corroborated by a probability plot (Fig. 7) with an R^2^ value of 0.9762^55^.

**Figure 8:**
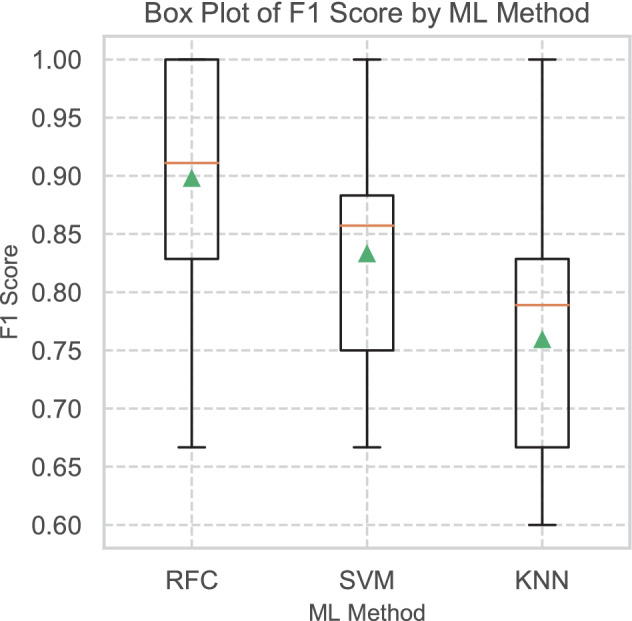
Box Plot of F1 Scores for each model, showing good equality of variances.

A Levene’s test of homogeneity^55^ demonstrated equality of variance between the models (0.43, p = 0.65) and is supported visually in Fig. 8.

Further post-hoc tests (Tukey Honestly Significant Difference (HSD), and Dunn-Šidák tests) showed there is a significant difference between RFC and KNN, but no significant difference between RFC and SVM, or between SVM and KNN.

### Assessing the value of collecting more samples

**Figure 9:**
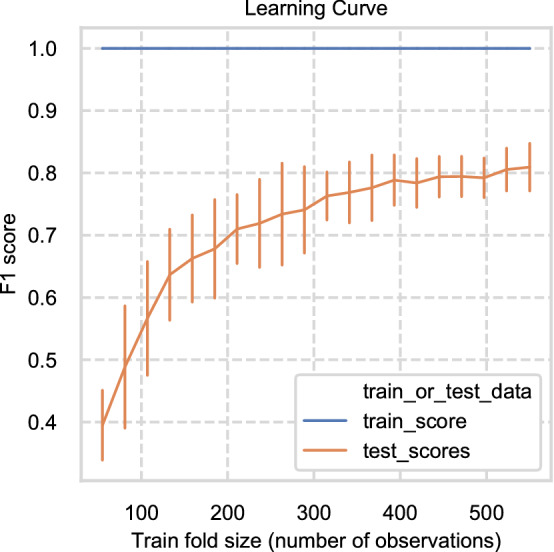
Learning curve shows F1 score for train and test data against number of observations in training data.

Given the results of the class performances, the next step was to assess whether acquiring more samples would improve overall model performance. Acquiring more data can increase the predictive performance of machine learning classifiers. To assess this we used a learning curve^29^, using several Random Forest classifiers built using 1000 trees and evaluated on increasingly larger training datasets. Each model was evaluated by stratified 10-fold cross-validation with F1 scores. The evaluation metric, the F1 score was then plotted as a function of training dataset size (Fig. 9), with the shape and gradient of the curve indicative of the value of training on more observations. The learning curve shows a rise in F1 score as the training data increased in size from 100 to 300 samples. The rate of increase then decreases above 300, with the gradient flattening towards 600 observations. This indicates that acquiring more observations may not necessarily increase the overall accuracy of a Random Forest Classifier.

There are several possible explanations for this flattening, including the general similarity of flint, limitations in the detection limits and noise associated with LA-ICP-MS, noise in the data between sample sites limiting the ability to distinguish them, and problems in the grouping of samples from different stratigraphic contexts at the sample sites because of too few samples per flint band. It is likely to be a combination of factors and further exploratory data analysis, laser and instrumental optimisation, or comparison with data from ICP-MS may inform on this.

### Assessing improving individual class size performance

In addition to assessing the value of collecting more samples overall, the next issue to be evaluated was whether the number of analyses per individual geological sample site would improve their classification performance. This issue would be particularly useful for the superficial geological deposit samples analysed in the authors’ previous research^21^, but as discussed above, due to the poor performance of these sites, they were not included in the evaluation of the machine learning techniques. This aspect of sampling is important as machine learning classifiers can underperform when classes have unbalanced proportions^56,57^. As a result, understanding whether acquiring more samples would increase model performance can guide future sampling efforts and give insight into the future potential for overall model performance.

**Figure 10:**
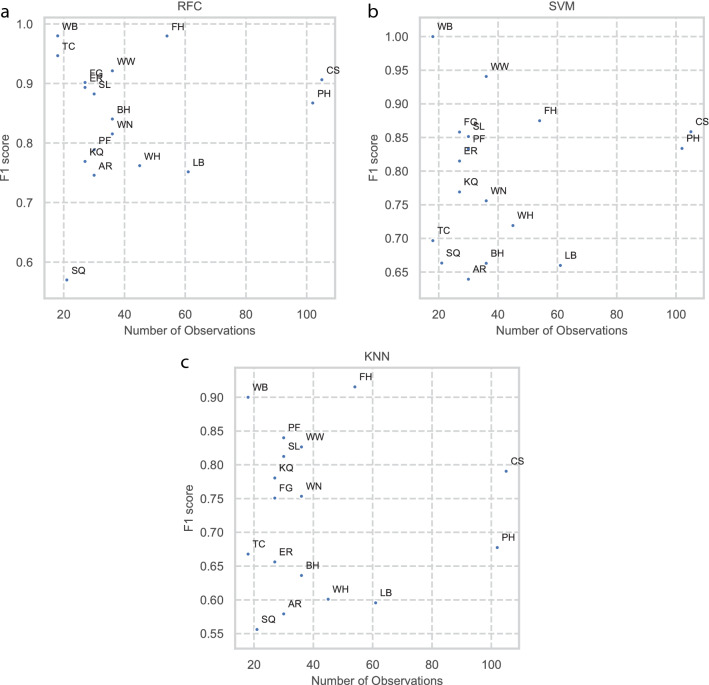
Graphical analysis of effect of class imbalance of class F1 scores. Scatterplots showing relationship between class F1 score and number of observations in the class for Random Forest Classifier (**a**), Support Vector Machine (**b**) and K nearest neighbour (**c**).

To assess class size performance, class-specific F1 scores were plotted against class sample size (Fig. 10a–c). In all models there was a positive correlation between class-specific F1 score and class sample size. If the bedrock sample sites CS and PH were treated as outliers there would be a strong positive correlation. Regardless, both CS and PH indicate strong performance in all three models, further supporting this interpretation. Together, these results suggest that acquiring additional samples from underrepresented bedrock sites would increase their individual class-specific F1 scores. Consequently, this would increase the F1 score of the models overall.

## Discussion

The research and methodology presented here demonstrate a robust means to evaluate machine learning techniques towards archaeological lithic sourcing and importantly includes a means of identifying outlier artefacts or analyses. The results show the viability of using machine learning techniques to classify flint in Britain at scale, with Random Forest showing the greatest overall potential, followed closely by Support Vector Machines.

The assessment of whether collecting more samples would improve overall performance indicates that this would likely not be the case, however it is likely greater sampling would help individual geological sites which performed poorly. On this first issue, there are likely several factors involved; including the natural variation within flint samples, any residual effect of noise from using LA-ICP-MS, any calibration bias issues, as well as the configuration of the classes generated prior to modelling. While the first of these represents a natural limit to differentiating between geological locations, refinement of the use of LA-ICP-MS and calibration protocols using a more appropriate matrix-matched reference material, or further comparison with solution ICP-MS may assist in improving analytical results. Lastly, further sampling and reconfiguration of the classes created prior to modelling to better represent the stratigraphic separation between samples is likely to assist with finer discrimination.

The feature selection and importance ranking conducted in this paper have broader geographic implications, highlighting similarities in the most predictive elements with research on the continent^20,47,49^. This apparent correlation warrants further attention, with the potential to help establish continent-scale sourcing studies on flint if proven, in much the same way as has been done for other lithic materials previously ^58–60^.

Returning to a national scale, the results presented here corroborate with previous research that flint from different locations and geologies can be reliably differentiated^44–49,61,62^, albeit with issues remaining. The results of this paper however show significantly greater separation of geological samples of flint than previously achieved through the greater identification of specific locations This can be compared most recently with the recent work of Bradley et al.^49,62^, who achieved broad, regional separation, as well as modest differentiation of sites, but whose work was hampered by more limited sampling and the use of a less powerful statistical technique. The increased spatial resolution gained through the greater number of sampling sites, and the greater differentiation between them seen in this project will be most impactful towards generating more specific and localised narratives of the prehistoric past^63–65^, and this is more apparent towards helping to evidence the procurement, movement and exchange of materials previously unknown. While the methodology here is applicable for prehistoric research more broadly, it may be most keenly felt within Mesolithic Studies, where a concerted effort by contemporary researchers is being made to move on from the abstract modelling and generalisation of previous generations, to more nuanced and specific social histories of the period^66–68^. For the Neolithic, this increased resolution towards the procurement of flint may perhaps place the distribution, trade, and exchange of objects such as axes^58,59,69,70^ in greater context, as well as help towards a more granular understanding of mobility for the period^71^.

## Conclusion

The results presented here demonstrate a robust machine learning pipeline for archaeological lithic sourcing, with the important addition of outlier detection. This last issue importantly removes the false positive assignment of artefacts to geological sample sites to which they do not belong. Overall, Random Forest performed the strongest, with an average 85% classification rate (measured through F1 scores) compared with Support Vector Machines and K-Nearest Neighbour. Analysis of these results through ANOVA revealed a significant difference between Random Forest and K-Nearest Neighbour.

The results of the class abundance against F1-score analyses demonstrates that class-specific performances will likely improve with greater sampling at geological sample sites with currently low numbers, with the caveat that, as seen in the learning curve, this will generate diminishing returns in increasing the overall accuracy of the models. Despite this last point, the results establish a clear basis for conducting future research and further sampling to aid in the sourcing of flint artefacts in Britain.

The methodology developed here demonstrates far greater spatial and stratigraphic separation of geological samples of flint in Britain than previously possible, suggesting that further isolation of sources may be possible with extended sampling. For bedrock geological sampling, it is likely that further refinement of the classes used to separate the data, such as accounting for stratigraphic differences, will likely aid future efforts, as well as broadening the number of sampling locations generally to increase spatial resolution. With regards to the superficial geological sampling data, it is likely that increased sampling at underperforming sites, and the use of other physical properties of the raw materials, such as colour and inclusions (as advocated by Brandl et al.^20^ in their multi-layered (MLA) approach) will aid in these sites being used. While the results here are promising, the scale of future work suggests the need for multiple studies focussing on the different aspects of this subject, including sampling, geochemical data analysis, refinement of the LA-ICP-MS analytical protocols, and further improvements to the subsequent data science methodology. The archaeological implications, however, are that the approach produced here may greatly assist in the location and identification of procurement sites and quarries in Britain when combined with geological mapping, survey, and reconnaissance.

## Supplementary Information

Supplementary Information.

## Data Availability

The data, code, and further details can be found on the primary author’s GitHub repository, available at: https://github.com/Spelaeo123.

## References

[1] Andrefsky W (2009). The analysis of stone tool procurement, production, and maintenance. J. Archaeol. Res..

[2] Odell, G. H. *Lithic Analysis*. (Springer, 2003).

[3] Dillian, C. D. & Renfrew, L. Twenty-five years on the cutting edge of obsidian studies: selected readings from the IAOS bulletin.

[4] Shackley, M. S. *Obsidian: geology and archaeology in the North American Southwest*. (University of Arizona Press, 2005).

[5] Cann JR, Renfrew C (1964). The characterization of obsidian and its application to the Mediterranean Region. Proc. Prehist. Soc..

[6] Andrefsky W (1994). Raw-material availability and the organization of technology. Am. Antiq..

[7] Odell GH (2001). Stone tool research at the end of the millenium: classification, function, and behaviour. J. Archaeol. Res..

[8] Boivin, N. & Owoc, M. A. *Soils, stones and symbols: cultural perceptions of the mineral world*. (Routledge, 2004).

[9] Freund KP (2013). An assessment of the current applications and future directions of obsidian sourcing studies in archaeological research. Archaeometry.

[10] Weigand, P., Harbottle, G. & Sayre, E. V. Turquoise sources and source analysis: Mesoamerica and the Southwestern U.S.A. in *Exchange Systems in Prehistory* (eds. Earle, T. K. & Ericson, J. E.) 15–34 (Academic Press, 1977).

[11] Parcak, S. *Satellite Remote Sensing for Archaeology*. (Routledge, 2009).

[12] Nilsson, A. *Predicting the archaeological landscape archeological density estimation around the Ostlänken railroad corridor predicting the archaeological landscape*. (2016).

[13] Roalkvam I (2020). Algorithmic classification and statistical modelling of coastal settlement patterns in mesolithic South-Eastern Norway. J. Comput. Appl. Archaeol..

[14] Anichini F (2020). Developing the ArchAIDE application: a digital workflow for identifying, organising and sharing archaeological pottery using automated image recognition. Internet Archaeol..

[15] Hörr, C., Lindinger, E. & Brunnett, G. Machine learning based typology development in archaeology. *J. Comput. Cult. Herit.* **7** (2014).

[16] Davidsson, P. Coin classification using a novel technique for learning characteristic decision trees by controlling the degree of generalization. In *Ninth International Conference on Industrial & Engineering Applications of Artificial Intelligence* (eds. Tanaka, T., Ohsuga, S. & Ali, M.) 403–412 (Gordon and Breach Science Publishers, 1996).

[17] Karasik, A., Sharon, I., Smilansky, U. & Gilboa, A. Typology and classification of ceramics based on curvature analysis. In *Computer Applications and Quantitative Methods in Archaeology 2003* (eds. Ausserer, K. F., Börner, W., Goriany, M. & Karlhuber-Vöckl, L.) 472–475. (Archaeopress, 2004).

[18] Maaten, L. van der, Boon, P., Lange, G., Paijmans, H. & Postma, E. Computer vision and machine learning for archaeology. In *Digital Discovery. Exploring New Frontiers in Human Heritage. CAA2006. Computer Applications and Quantitative Methods in Archaeology. Proceedings of the 34th Conference, Fargo, United States, April 2006.* (eds. Clark, J. T. & Hagemeister, E. M.) 476–482 (Archaeolingua, 2007).

[19] Flores FC (2019). Computer algorithm for archaeological projectile points automatic classification. J. Comput. Cult. Herit..

[20] Brandl, M. *et al. A multi-technique analytical approach to sourcing Scandinavian flint: Provenance of ballast flint from the shipwreck “Leirvigen 1”, Norway*. *PLoS ONE* **13**, (2018). 10.1371/journal.pone.0200647 PMC6082525 30089119

[21] Elliot, T. The mesolithic in the marches: geochemical lithic sourcing in the lower Wye Valley. (University of Worcester, 2019).

[22] Mcalister A (2019). On provenance studies of New Zealand obsidians: A pXRF-based geochemical reference dataset and a review of analytical methods. Archaeol. Ocean..

[23] Moreau L (2018). First geochemical ‘fingerprinting’ of Balkan and Prut flint from Palaeolithic Romania: potentials, limitations and future directions. Archaeometry.

[24] Egeland CP (2019). Geochemical and physical characterization of lithic raw materials in the Olduvai Basin, Tanzania. Quat. Int..

[25] Moreau L (2016). Geochemical sourcing of flint artifacts from western Belgium and the German Rhineland: testing hypotheses on Gravettian period mobility and raw material economy. Geoarchaeology.

[26] Barclay GJ, Brophy K, Barclay GJ (2020). ‘ A veritable chauvinism of prehistory ’: nationalist prehistories and the ‘ British ’ late Neolithic mythos prehistories and the ‘ British ’ late Neolithic mythos. Archaeol. J..

[27] James, G., Witten, D., Hastie, T. & Tibshirani, R. *An Introduction to Statistical Learning: with Applications in R*. (Springer, 2017).

[28] Oliphant, T. E. *A guide to NumPy*. (Trelgol Publishing, 2006).

[29] Pedregosa F (2011). Scikit-learn: machine learning in python. J. Mach. Learn. Res..

[30] McKinney, W. Data structures for statistical computing in python. In *Proceedings of the 9th Python in Science Conference* (eds. van der Walt, S. & Millman, J.) 51–56 (2010).

[31] Hunter JD (2007). Matplotlib: a 2D graphics environment. Comput. Sci. Eng..

[32] Waskom, M. *et al.* mwaskom/seaborn: v0.11.1 (December 2020). (2020). 10.5281/ZENODO.4379347

[33] Carpenter, J. M. Swifter 0.260. (2018). https://pypi.org/project/swifter/#description. (Accessed: 20th November 2018)

[34] Lemaître G, Nogueira F, Aridas CK (2017). Imbalanced-learn: a python toolbox to tackle the curse of imbalanced datasets in machine learning. J. Mach. Learn. Res..

[35] Hand D, Christen P (2018). A note on using the F-measure for evaluating record linkage algorithms. Stat. Comput..

[36] Haibo H, Garcia EA (2009). Learning from imbalanced data. IEEE Trans. Knowl. Data Eng..

[37] Jolliffe IT, Cadima J (2016). Principal component analysis: a review and recent developments. Philos. Trans. R. Soc. A Math. Phys. Eng. Sci..

[38] Mai Q (2013). A review of discriminant analysis in high dimensions. Wiley Interdiscip. Rev. Comput. Stat..

[39] van der Maaten L, Hinton G (2008). Visualizing data using t-SNE. J. Mach. Learn. Res..

[40] Breunig MM, Kriegel H-P, Ng RT, Sander J (2000). LOF: identifying density-based local outliers. ACM SIGMOD Rec..

[41] Guyon I, Weston J, Barnhill S, Vapnik V (2002). Gene selection for cancer classification using support vector machines. Mach. Learn..

[42] Breiman L (2001). Random forests. Mach. Learn..

[43] Kohavi, R. A study of cross-validation and bootstrap for accuracy estimation and model selection. *Int. Jt. Conf. Artif. Intell.* 0–6 (1995).

[44] Sieveking GDG, Craddock PT, Hughes MJ, Bush PR, Ferguson J (1970). Characterisation of prehistoric flint mine products. Nature.

[45] Sieveking GDG (1972). Prehistoric flint mines and their identification as sources of raw material. Archaeometry.

[46] Thompson, M., Bush, P. R. & Ferguson, J. The Analysis of flint by Inductively Coupled Plasma Atomic Emission Spectrometry, As a Method for Source Determination. in *The scientific study of flint and chert: Proceedings of the fourth international flint symposium held at Brighton Polytechnic 10–15 April 1983* (eds. Sieveking, G. D. G. & Hart, M. B.) 243–248 (Cambridge University Press, 1986).

[47] Rockman, M. Landscape Learning in the Late Glacial Recolonization of Britain. (University of Tucson, 2003).

[48] Pettitt P, Rockman M, Chenery S (2012). The British Final Magdalenian: Society, settlement and raw material movements revealed through LA-ICP-MS trace element analysis of diagnostic artefacts. Quat. Int..

[49] Bradley S, Cummings V, Baker MJ (2020). Sources of flint in Britain and Ireland: a quantitative assessment of geochemical characterisation using acid digestion inductively coupled plasma-mass spectrometry (ICP-MS). J. Archaeol. Sci. Rep..

[50] Mortimore, R. N., Wood, C. J. & Gallois, R. W. British upper cretaceous stratigraphy. *Geol. Conserv. Rev. Ser. No. 23, Jt. Nat. Conserv. Committee, Peterbrgh.* **23**, 558 (2001).

[51] Python for Data Science. One-way ANOVA. *Python for Data Science* (2021). https://www.pythonfordatascience.org/anova-python/. (Accessed: 1st March 2021)

[52] Seabold, S. & Perktold, J. Statsmodels: Econometric and Statistical Modeling with Python. in *Proceedings of the 9th Python in Science Conference.* 92–96 (2010). 10.25080/Majora-92bf1922-011

[53] Virtanen P (2020). SciPy 1.0: fundamental algorithms for scientific computing in Python. Nat. Methods.

[54] Shapiro SS, Wilk MB (1965). An analysis of variance test for normality (complete samples). Biometrika.

[55] NIST/SEMATECH. NIST/SEMATECH e-Handbook of Statistical Methods. (2012). 10.18434/M32189

[56] Japkowicz N, Stephen S (2002). The class imbalance problem: a systematic study. Intell. Data Anal..

[57] Shultz, T. R. *et al.* Class Imbalance Problem. in *Encyclopedia of Machine Learning* 171–171 (Springer US, 2011). doi:10.1007/978-0-387-30164-8_110

[58] Clough, T. H. M. & Cummins, W. A. *Stone Axe Studies: Archaeological, Petrological, Experimental and Ethnographic, CBA Research Report No. 23*. (Council for British Archaeology, 1979). doi:10.1002/gea.3340050108

[59] Davis, V. & Edmonds, M. R. *Stone Axe Studies III*. (Oxbow Books, 2011).

[60] Pétrequin, P. *et al.* Neolithic Alpine axeheads, from the Continent to Great Britain, the Isle of Man and Ireland. In *Between Foraging and Farming: an Extended Broad Spectrum of Papers Presented to Leendert Louwe Kooijmans* (eds. Fokkens, H. et al.) 261–79 (Leiden University, 2008).

[61] Hughes RE, Högberg A, Olausson D (2010). Sourcing flint from Sweden and Denmark. J. Nord. Archaeol. Sci..

[62] Bradley, S. Archaeological and geochemical investigation of flint sources in Britain and Ireland. (University of Central Lancashire, 2017).

[63] Ingold, T. Taking taskscape to task. In *Forms of Dwelling: 20 Years of the Taskscapes in Archaeology* (eds. Rajala, U. & Mills, P.) 16–27 (Oxbow Books, 2017).

[64] Nyland, A. J. Materialised taskscapes? Mesolithic lithic procurement in Southern Norway. In *Forms of Dwelling: 20 Years of the Taskscapes in Archaeology* (eds. Rajala, U. & Mills, P.) 125–150 (Oxbow, 2017).

[65] Edmonds, M. R. Taskscape, technology and tradition. *Leiden. Analecta Praehist.* **29**, (1997).

[66] Warren GM (2018). From moments to histories: a social archaeology of the mesolithic?. J. World Prehistory.

[67] Milner, N. & Woodman, P. *Mesolithic studies at the beginning of the 21st century*. (Oxbow Books, 2005).

[68] Conneller, C. & Warren, G. *Mesolithic Britain and Ireland: New Approaches*. (Tempus Publishing, 2006).

[69] Schauer P (2020). British neolithic axehead distributions and their implications. J. Archaeol. Method Theory.

[70] Edmonds MR (1997). Taskscape, technology, tradition. Analecta Praehist. Leiden..

[71] Leary, J. & Kador, T. Movement and mobility in the Neolithic. in *Moving on in Neolithic studies : Understanding mobile lives: Neolithic Studies Group Seminar Papers 14* (eds. Leary, J. & Kador, T.) (Oxbow Books, 2016).

