# Publisher Correction: Evaluating machine learning techniques for archaeological lithic sourcing: a case study of flint in Britain

**DOI:** 10.1038/s41598-021-02402-z

**Published:** 2021-11-22

**Authors:** Tom Elliot, Robert Morse, Duane Smythe, Ashley Norris

**Affiliations:** 1 Department of Archaeology, Classics and Egyptology, University of Liverpool, 12-14 Abercromby Square, Liverpool, L69 7WZ UK; 2 Intelligent Ultrasound, Floor 6A, Hodge House, 114-116 St Mary Street, Cardiff, CF10 1DY UK; 3 Department of Earth Sciences, South Parks Road, Oxford, OX1 3AN UK; 4 Norris Scientific, PO Box 812, Kingston, TAS 7050 Australia

Correction to: *Scientific Reports* 10.1038/s41598-021-87834-3, published online 13 May 2021

The original version of this Article contained errors in the legends of Figure 8 and 9.

The legend of Figure 8:

“Learning curve shows F1 score for train and test data against number of observations in training data.”

now reads:

“Box Plot of F1 Scores for each model, showing good equality of variances.”

The legend of Figure 9:

“Evaluation of data (**a**) Scatter plot showing F1 scores for models built on features selected by recursive feature elimination. (**b**) Learning curve shows F1 score for train and test data against number of observations in training data.”

now reads:

“Learning curve shows F1 score for train and test data against number of observations in training data.”

The original Article has been corrected.

