# Supplementary material for: Evaluating machine learning techniques for archaeological lithic sourcing: a case study of flint in Britain
Source: Sci Rep. 2021 May 13;11:10197. doi: 10.1038/s41598-021-87834-3 (PMC8119680; doi:10.1038/s41598-021-87834-3)
Supplement: Supplementary file 1 — Supplementary Information. [file 41598_2021_87834_MOESM1_ESM.docx]

# Supplementary Information

The selection of hyperparameters were optimised by random grid search and are presented in Table 6 below.

Table 6: Table of Hyperparameters for each Machine Learning Technique

| ML Technique | Hyperparameters |
| --- | --- |
| Random Forest Classifier: | n_estimators = [2, 5]  max_depth = [8, 12]  min_samples_split = [2, 4]  min_samples_leaf = [1, 3] |
| Support Vector Machine: | C = [0.01, 0.1, 1, 10, 100],  class_weight = ['balanced', None],  gamma = [0.001, 0.01, 0.1, 1, 10] |
| KNN: | n_neighbors = [4, 6, 8, 10, 12, 14]  weights = ['uniform', 'distance'] |
